# Dynamic response force control of electrohydraulic servo actuator of active suspension based on intelligent optimization algorithm

**DOI:** 10.1371/journal.pone.0323066

**Published:** 2025-06-10

**Authors:** Qinghe Guo, Mengchao Wang, Renjun Liu, Yurong Chen, Shenghuai Wang, Hongxia Wang

**Affiliations:** 1 School of Mechanical Engineering, Hubei University of Automotive Technology, Shiyan, Hubei, China; 2 Key Laboratory of Automotive Power Train and Electronics, Hubei University of Automotive Technology, Shiyan, Hubei, China; 3 School of Art and Design, Hubei University of Automotive Technology, Shiyan, Hubei, China; Anurag University, INDIA

## Abstract

Traditional PID control faces challenges in addressing parameter uncertainty and nonlinearity in active suspension electrohydraulic servo actuators, leading to suboptimal performance. To address these challenges, a fractional-order PID (FOPID) controller optimization method based on the Multi-Strategy Improved Beluga Whale Optimization (MSIBWO) algorithm is proposed. Simulation results in MATLAB/Simulink demonstrate that the MSIBWO-FOPID controller significantly outperforms traditional PID and BWO-FOPID controllers in force tracking and robustness. For step input, the rise time and the root mean square error(RMSE) are reduced by 66.7% and 70.3%, respectively, compared to BWO-FOPID. For sine inputs, the system achieves better disturbance rejection and higher precision. Using a half-car model, the MSIBWO-FOPID controller improves ride comfort significantly. Under random road excitation, the RMSE values of the vehicle body’s vertical acceleration and pitch angle acceleration are reduced by 51.7% and 13.1%, respectively, compared to passive suspension, outperforming both PID and BWO-FOPID controllers.

## 1 Introduction

In hydraulic active suspension systems, the electrohydraulic servo actuator is the core component for outputting active control force [1]. Its efficient force-tracking performance is crucial for achieving optimal ride comfort and handling stability in active suspension vehicles. However, the force-tracking accuracy of electrohydraulic servo actuators is significantly affected by parameter uncertainties, external disturbances, and system nonlinearities during actual control processes. To address these challenges, it is essential to develop a controller capable of overcoming parameter uncertainties and nonlinear behaviors to ensure precise force-tracking performance and enhance the overall system performance [2].

In recent years, significant research efforts have been devoted to improving the control performance of electrohydraulic servo systems. Numerous advanced control strategies have been proposed, including PID control [3, 4], sliding mode control [5], active disturbance rejection control [6], and composite control strategies [7, 8]. Among these, PID controllers are widely adopted in various control systems, including active suspension applications, due to their simplicity and ease of implementation. However, the inherent nonlinearities and parameter uncertainties of electrohydraulic servo actuators often render traditional PID control inadequate for meeting the precise control demands of active suspension systems [9].

To address these challenges, fractional-order PID (FOPID) controllers, which extend conventional PID by incorporating fractional calculus, have garnered significant attention. FOPID controllers offer superior performance in handling complex nonlinear systems [10, 11]. For instance, Ansarian *et al*. [10] developed a FOPID controller for nonlinear drone systems, achieving notable improvements in dynamic response and stability. Hasan *et al*. [11] addressed the challenges of nonlinearity and time variance in autonomous underwater vehicles (AUVs) by designing a FOPID controller, with experimental results confirming its effectiveness. For the magnetic levitation systems, Mughees *et al*. [12] applied a FOPID strategy method that effectively enhanced position control accuracy and stability. Due to the inherent nonlinearity and complexity of pneumatic control valve systems, traditional PID controllers struggle to effectively address these challenges. He *et al*. [13] introduced a FOPID controller, and experimental results demonstrated that the FOPID controller exhibited superior dynamic performance. Despite these advantages, the FOPID controller introduces additional tuning parameters compared to traditional PID controllers, making the tuning process significantly more complex. The control performance of FOPID heavily depends on precise parameter selection, which remains a key research challenge [14]. This motivates the exploration of advanced optimization algorithms to systematically and efficiently tune FOPID parameters for improved control performance in nonlinear systems.

With the rapid development of artificial intelligence technologies, researchers have increasingly formulated the parameter tuning problem as an optimization problem [15–17]. In the field of nuclear reactor control, Rafiei *et al*. [18] applied the FOPID controller for load-following control, optimizing the controller parameters using the genetic algorithm (GA). Simulation results indicated that the GA-FOPID controller achieved satisfactory performance. However, studies have shown that traditional optimization algorithms, such as particle swarm optimization (PSO) algorithm and genetic algorithm, have inferior overall performance compared to novel optimization algorithms [19]. Consequently, many researchers have employed advanced optimization algorithms for controller parameter tuning. For example, Mazumdar *et al*. [20] applied the Grey Wolf Optimization (GWO) algorithm to optimize FOPID parameters, achieving improved integration in power network systems and validating the effectiveness of the proposed controller. Similarly, Kommula *et al*. [21] introduced an intelligent FOPID controller for brushless motors, which demonstrated faster system response compared to traditional PID controller. Ayas *et al*. [22] utilized the Sine-Cosine Algorithm (SCA) to optimize FOPID parameters, significantly enhancing the performance of Automatic Voltage Regulator (AVR) systems, as shown through experimental results. Noman *et al*. [23] introduced the marine predator optimization algorithm (MPA) for FOPID parameter tuning, effectively improving the interference resistance and robustness of AVR systems. El-Bahay *et al*. [24] designed a FOPID controller based on the coot optimization algorithm (COA) for a two-area power system. Compared to traditional PID controllers and other optimization algorithms, the COA-FOPID controller exhibited the best control performance.

Although thse studies have made progress in controller parameter optimization, their performance is limited in addressing specific problems. While these approaches have improved the efficiency of parameter tuning, they often suffer from premature convergence and being trapped in local optima when dealing with high-dimensional and nonlinear systems. To address these issues, this paper proposes a multi-strategy improved beluga whale optimization algorithm (MSIBWO) aimed at enhancing the speed and accuracy of solving optimization problems. The MSIBWO algorithm employs a population diversity preservation strategy to ensure uniform distribution of the initial population in the solution space, thereby improving the quality of the initial population. By introducing a position update operator, the algorithm enhances global search capability in the early stages, effectively avoiding premature convergence and local optima. Additionally, a dynamic adaptive weight mechanism is used to adjust the search step size dynamically, balancing global and local search capabilities. The combination of these strategies enables the MSIBWO algorithm to perform exceptionally well in handling high-dimensional and nonlinear optimization problems. Given the above discussion, the contributions of this paper are as follows:

- A multi-strategy improved beluga whale optimization (MSIBWO) algorithm is proposed. By incorporating a population diversity preservation strategy, a position update operator, and dynamic adaptive weight, the algorithm significantly improves the speed and accuracy of solving optimization problems. Compared to existing optimization algorithms, the MSIBWO algorithm demonstrates stronger global search capability and higher convergence precision when addressing high-dimensional and nonlinear systems.
- Intelligent optimization control strategies are designed using the electrohydraulic servo actuator model and the half-car model as research subjects, and their effectiveness is verified. Experimental results show that the FOPID controller based on the MSIBWO algorithm outperforms traditional PID controllers and BWO-FOPID controllers in terms of force-tracking accuracy and system stability.

The remainder of this paper is organized as follows: Section 2 introduces the MSIBWO algorithm, including its design and improvement strategies. Section 3 establishes the mathematical modeling of the electrohydraulic servo actuator and the half-vehicle system. Section 4 describes the design and implementation of the MSIBWO-FOPID controller. Section 5 validates the effectiveness of the proposed strategies. Finally, Section 6 concludes with a summary and outlook.

## 2 Improved beluga whale optimization algorithm

The BWO algorithm, inspired by the biological behaviors of whales, mimics three key behaviors: swimming, foraging, and breaching, which correspond to the exploration, exploitation, and whale fall phases within the algorithm [25]. Despite its merits, the BWO algorithm is hindered by its poor initialization quality and difficulty in balancing exploration and exploitation. These drawbacks often lead to premature convergence, trapping the algorithm in local optima, and result in slow convergence rates. To overcome these limitations, The MSIBWO algorithm is proposed.

### 2.1 Multi-strategy improved beluga whale optimization algorithm

#### 2.1.1 Improving the initialization phase.

The initialization of the BWO algorithm often lacks diversity and fails to adequately cover the entire solution space. To address this, we introduce chaotic mapping using the Tent map [26] to initialize whale positions. This ensures a more uniform and diverse distribution of positions across the solution space. The Tent chaotic map is chosen over other widely used chaotic maps, such as the Logistic or Piecewise maps, due to its superior traversal and uniformity properties, which enhance the exploration phase of the algorithm [27, 28].

Furthermore, to enhance the quality of the initialized whale positions, we incorporate opposition-based learning (OBL) [29], which seeks to generate solutions closer to the global optimum by simultaneously considering the opposite positions of generated solutions. This dual-strategy approach combining chaotic mapping and OBL improves the diversity of the initial population, thereby strengthening the algorithm’s global search capability and accelerating convergence. The improved initialization formula for whale positions using the Tent chaotic map is as follows:

$$\left\{ \begin{array}{cc} X_{i,j}^{T+1}=\lambda X_{i,j}^{T}, & X_{n}\in [lb,\alpha ]\\ \lambda (1-X_{i,j}^{T}), & X_{n}\in [\alpha ,ub] \end{array}\right.$$ (1)

where $X_{i,j}^{T}$ represents the position of the whale corresponding to the *ith* row and *jth* column. Here, λ represents a system parameter, while *ub*,*lb* represent the upper and lower bounds for initializing the whale’s position.

The whale positions obtained through Eq 1 are denoted as *X*_*i*,*j*_. To enhance the quality of the initial whale position distribution using the opposition-based learning (OBL) strategy, the opposite whale position ${\overset{˘}{X}}_{i,j}$ is calculated using Eq 2.

$${\overset{˘}{X}}_{i,j}=ub+lb-X_{i,j}$$ (2)

Combining the *n* sets of whale positions obtained from the Tent chaotic mapping and the *n* sets of OBL derived opposite whale positions, we obtain the overall whale positions, denoted as $\left[X_{i,j};{\overset{˘}{X}}_{i,j}\right]$. These positions are sorted based on their corresponding fitness values. Through a principle of survival of the fittest, *n* sets of initialized whale positions are determined, denoted as ***X***, and *n* is the population size of beluga whales.

#### 2.1.2 Improving the exploration phase.

The exploration and exploitation behavior of the MSIBWO algorithm is controlled by the balance factor *B*_*f*_. Specifically, when *B*_*f*_>0.5, the algorithm prioritizes exploration, while $B_{f}\leq 0.5$ shifts the focus to exploitation. The mathematical model is as follows:

$$B_{f}=B_{0}\left(1-\frac{T}{2T_{\max}}\right)$$ (3)

where *B*_0_ represents a random number uniformly distributed in the interval (0, 1), *T* represents the current iteration number, and $T_{\text{max}}$ signifies the maximum number of iterations.

We introduce the position update operator from the slap swarm algorithm [30], which enhances the global and local search capabilities of the BWO algorithm. To further strengthen the exploration phase in the early iterations, we modify the slap swarm-inspired operator to improve the algorithm’s search range and diversity. This modification ensures that the algorithm can thoroughly explore the solution space before transitioning to the refinement phase. By balancing the range and precision of the search process, the proposed approach enhances the robustness of the global search during the initial stages of optimization. The expression for the position update operator is as follows:

$$E=\frac{r_{1}[(ub-lb)r_{2}+lb]}{(1+r_{3})ub}$$ (4)

After incorporating *E* into the BWO optimization algorithm, the improved formula for updating the whale positions during the exploration phase Eq 5 is as follows:

$$\left\{ \begin{array}{c} X_{i,j}^{T+1}=X_{i,p_{j}}^{T}+\left(X_{r,p_{1}}^{T}-X_{i,p_{j}}^{T}\right)\left(1+E\right)\sin\left(2\pi r_{4}\right),\;j=even\\ X_{i,j}^{T+1}=X_{i,p_{j}}^{T}+\left(X_{r,p_{1}}^{T}-X_{i,p_{j}}^{T}\right)\left(1+E\right)\cos\left(2\pi r_{4}\right),\;j=odd \end{array}\right.$$ (5)

where $X_{i,j}^{T+1}$ represents the new position updated iteratively; *r* denotes a random whale; *p*_*j*_ signifies a random dimension, which is three dimensions for PID parameters;$r_{1},r_{2},r_{3}$, and *r*_4_ are random number within the range of 0 to 1.

#### 2.1.3 Improving the exploitation phase.

In the exploitation phase, the BWO algorithm utilizes Levy flights to enhance convergence by adjusting step sizes, thereby balancing global and local search capabilities. Levy flights, which are characterized by long jumps interspersed with short, random movements, enable the algorithm to escape local optima and refine solutions in the vicinity of the global optimum.

To further optimize the exploitation phase, we introduce a dynamic adaptive weighting mechanism [31] that adjusts the algorithm’s focus as iterations progress. In the early stages, the adaptive weight gradually increases to emphasize global exploration. In later stages, the weight decreases to prioritize local search, ensuring faster convergence to the optimal solution. This dynamic adjustment creates a smooth transition between exploration and exploitation, significantly improving the algorithm’s ability to balance exploration diversity and exploitation efficiency. The formula for dynamic adaptive weighting is as follows:

$$\text{S}=\left\{ \begin{array}{c} 2e^{-6(T_{\max}-T)/T_{\max}},T\leq T_{\max}/2\\ 2e^{-6T/T_{\max}},T>T_{\max}/2 \end{array}\right.$$ (6)

After incorporating *S* into the BWO optimization algorithm, the improved formula for updating the whale positions during the exploitation phase Eq 7 is as follows:

$$\left\{ \begin{array}{c} X_{i}^{T+1}=r_{5}X_{\text{best}}^{T}-SX_{i}^{T}+C_{1}\cdot L_{F}\cdot \left(X_{r}^{T}-X_{i}^{T}\right)\\ C_{1}=2S\left(1-T/T_{\max}\right) \end{array}\right.$$ (7)

where $X_{i}^{T+1}$ represents the new position, $X_{\text{best}}^{T}$ denotes the current best position, $X_{i}^{T}$ represents the current position, $X_{r}^{T}$ stands for the current random position, *r*_5_ are random numbers within the range of 0 to 1. *C*_1_ denotes the random jump strength of Levy flights, where Levy flight is mathematically expressed as follows:

$$L_{F}=0.05\times \frac{u_{i}\times \sigma }{|v_{i}{|}^{1/\beta }}$$ (8)

where *u*_*i*_ and $v_{i}$ are normally distributed random numbers, the value of β is 1.5. The expression for σ is as follows:

$$\sigma ={\left(\frac{\Gamma \left(1+\beta \right)\times \sin\left(\pi \beta /2\right)}{\Gamma \left(\left(1+\beta \right)/2\right)\times \beta \times 2^{(\beta -1)/2}}\right)}^{1/\beta }$$ (9)

#### 2.1.4 The Whale fall phase.

The probability of a whale falling into the deep sea during migration, denoted as whale fall probability *W*_*f*_, is mathematically expressed as:

$$W_{f}=0.1-0.05\left(T/T_{\max}\right)$$ (10)

To keep the total number of whales in the population unchanged, the whale positions are updated using the current whale positions and the whale fall step size $X_{\text{step}}$.

$$\left\{ \begin{array}{c} X_{i}^{T+1}=r_{6}X_{i}^{T}-r_{7}X_{r}^{T}+r_{8}X_{step}\\ X_{step}=(ub-lb)\exp(-C_{2}\left(T/T_{\max}\right))\\ C_{2}=2W_{f}\times n \end{array}\right.$$ (11)

where $r_{6},r_{7}$, and *r*_8_ are random numbers uniformly distributed between 0 and 1, *C*_2_ represents the step factor.

### 2.2 The procedure of MSIBWO

**Fig 1:**
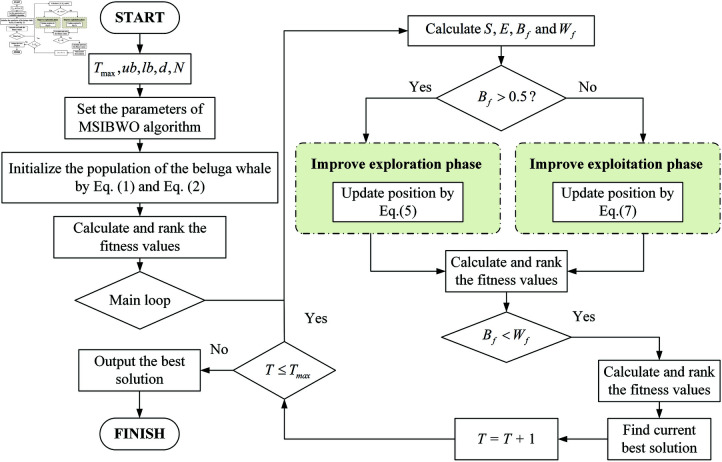
The flow chart of the MSIBWO algorithm.

To provide a clearer understanding of its operational principles, the flow chart of the MSIBWO algorithm is shown (see Fig 1). In order to describe the structure of the algorithm more clearly and intuitively, and to enhance readability, we have provided the pseudo-code of the algorithm. The corresponding pseudo-code (see Algorithm 1).

**Algorithm 1. The pseudo-code of MSIBWO algorithm**

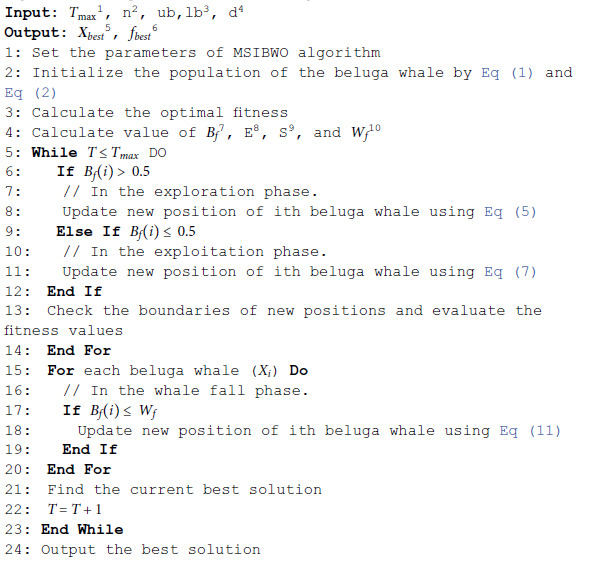



### 2.3 Performance testing of the algorithm

#### 2.3.1 Benchmark function test.

To evaluate the performance of the proposed MSIBWO algorithm, we conducted comparative tests against five other algorithms: BWO, GWO [32], WOA [33], AOA [34], SSA [35], and CPO [36]. The experiments were carried out on a 64-bit Windows 11 operating system running on an Intel(R) Core(TM) i7-12700H CPU @ 2.30GHz, utilizing MATLAB version 2021a. The benchmark functions used for testing are summarized in Table 1.: F1-F4 are unimodal benchmark functions designed to evaluate the algorithms’ convergence speed and optimization accuracy. F5-F8 are multimodal benchmark functions designed to assess the algorithms’ global search capabilities.

**Table 1:** Benchmark functions.

Function	Dimension	Range	${𝐟}_{min}$
$F_{1}(x)=\sum_{i=1}^{n}x_{i}^{2}$	30	[-100,100]	0
$F_{2}(x)=\sum_{i=1}^{n-1}\left[100{\left(x_{i+1}-x_{1}^{2}\right)}^{2}+{\left(x_{i}-1\right)}^{2}\right]$	30	[-30,30]	0
$F_{3}(x)=\sum_{i=1}^{n}{\left(\sum_{j-1}^{i}x_{j}\right)}^{2}$	30	[-100,100]	0
$F_{4}(x)=max\left\{\left|x_{i}\right|,1\leq i\leq n\right\}$	30	[-100,100]	0
$F_{5}(x)=-\sum_{i=1}^{n}\left(x_{i}sin\sqrt{\left|x_{i}\right|}\right)$	30	[-500,500]	-12569.4
$F_{6}(x)=-20\exp\left(-0.2\sqrt{\left(\sum_{i=1}^{n}{x_{i}}^{2}\right)/n}\right)-\exp\left(\left(\sum_{i=1}^{n}\cos(2\pi x_{i})\right)/n\right)+20+e$	30	[-32,32]	0
$F_{7}(x)=\sum_{i=1}^{11}{\left|a_{i}-\frac{x_{1}\left(b_{i}^{2}+b_{i}x_{2}\right)}{b_{i}^{2}+b_{i}x_{3}+x_{4}}\right|}^{2}$	4	[-5,5]	0.000308
$F_{8}(x)=-\sum_{i=1}^{5}{\left|\left(x_{i}-a_{i}\right){\left(x_{i}-a_{i}\right)}^{T}+c_{i}\right|}^{-1}$	4	[0,10]	-10.1532

The parameter settings for each algorithm are detailed in Table 2. To ensure a fair comparison, all algorithms were configured with the same population size of 30 and a maximum iteration count of 300. Each algorithm was tested 30 times on each benchmark function to account for stochastic variation, with results recorded for the minimum value(f_min_), average value, and standard deviation (Std) across these runs.

**Table 2:** The parameter settings for each algorithm.

Algorithm	Parameters	Value
MSIBWO	Minimun and maximum probability of whale fall	[0.05, 0.1]
BWO	Minimun and maximum probability of whale fall	[0.05, 0.1]
GWO	Minimun and maximum convergence constant	[0, 2]
WOA	The probability of spiral factor	0.51
AOA	Sensitive parameter Control parameter	$AOA_{\alpha }$ = 5, $AOA_{\mu }$ = 0.5
SSA	The proportion of number of sparrows in each stage	[PD, ST, SD] = [20%, 20%, 80%]
CPO	Aroma Concentration Factor	[0, 0.07]

Table 3 summarizes the results of the benchmark functions F1 to F8, with the best results highlighted in bold. The f_min_ and average value are used to evaluate the optimization capabilities of the algorithms, while the Std quantifies their robustness.

**Table 3:** Test results.

Function	Algorithm	${𝐟}_{min}$	Average	Std
*F*_1_(*x*)	MSIBWO	**0**	**0**	**0**
	BWO	8.78E-267	6.59E-256	**0**
	GWO	2.60E-29	1.36E-27	2.92E-27
	WOA	2.83E-86	2.42E-69	1.33E-68
	AOA	8.50E-146	2.00E-40	8.38E-40
	SSA	1.19E-247	1.38E-58	7.52E-58
	CPO	2.15E-224	2.84E-114	1.56E-113
*F*_2_(*x*)	MSIBWO	**0**	**0**	**0**
	BWO	7.70E-136	3.63E-131	8.01E-131
	GWO	1.88E-17	8.63E-17	7.27E-17
	WOA	2.11E-58	4.80E-51	1.50E-50
	AOA	**0**	**0**	**0**
	SSA	1.67E-285	4.25E-29	2.33E-28
	CPO	2.20E-113	8.96E-55	4.90E-54
*F*_3_(*x*)	MSIBWO	**0**	**0**	**0**
	BWO	2.09E-254	1.81E-240	0
	GWO	4.44E-09	8.19E-05	2.444E-04
	WOA	14200.59	40529.59	14606.537
	AOA	3.63E-144	0.003087	0.0087033
	SSA	5.11E-101	5.93E-27	2.76E-26
	CPO	1.35E-189	1.20E-111	6.58E-111
*F*_4_(*x*)	MSIBWO	**0**	**0**	**0**
	BWO	4.66E-133	1.91E-126	9.48E-126
	GWO	1.32E-07	6.03E-07	3.97E-07
	WOA	0.022459	48.809296	29.76188
	AOA	5.56E-60	0.0221772	0.0229085
	SSA	6.72E-137	2.64E-27	1.43E-26
	CPO	6.68E-106	1.77E-51	9.65E-51
*F*_5_(*x*)	MSIBWO	**-12569.486**	**-12569.486**	**1.85009E-12**
	BWO	**-12569.486**	**-12569.486**	3.30345E-08
	GWO	-8061.641126	-6041.864508	930.5611284
	WOA	-12568.57472	-10627.92977	1636.14755
	AOA	-6510.891513	-5292.726563	548.689507
	SSA	-10019.99789	-8843.351087	608.7422529
	CPO	-6794.786159	-3802.867498	1544.018915
*F*_6_(*x*)	MSIBWO	**8.88E-16**	**8.88E-16**	**1E-31**
	BWO	**8.88E-16**	**8.88E-16**	**1E-31**
	GWO	7.54952E-14	1.02496E-13	1.95E-14
	WOA	8.88178E-16	4.79616E-15	3E-15
	AOA	**8.88E-16**	**8.88E-16**	**1E-31**
	SSA	**8.88E-16**	**8.88E-16**	**1E-31**
	CPO	**8.88E-16**	**8.88E-16**	**1E-31**
*F*_7_(*x*)	MSIBWO	**0.000308063**	**0.000324**	**2.29E-05**
	BWO	0.000312584	0.000361	5.12E-05
	GWO	0.000307507	0.004474	0.008086
	WOA	0.000307794	0.000885	0.000587
	AOA	0.000328832	0.023301	0.035447
	SSA	0.000307486	0.000379	0.000235
	CPO	0.000307655	0.000533	0.000866
*F*_8_(*x*)	MSIBWO	**-10.1531997**	**-10.1531994**	**7.85E-07**
	BWO	-10.1531944	-10.15053	0.0032193
	GWO	-10.1530384	-8.7569	2.6247473
	WOA	-10.1525399	-8.348906	2.603127
	AOA	-8.3466542	-3.677784	1.6546062
	SSA	**-10.1531997**	-8.623755	2.3761063
	CPO	-10.1531969	-10.15313167	7.28E-05

As shown in Table 3, for the unimodal benchmark functions (F1-F4), the MSIBWO algorithm achieves f_min_ and average value of 0, which are exactly equal to the optimal solutions of the test functions. This highlights the algorithm’s high precision in search capability. Additionally, the Std values are consistently 0, confirming the robustness of MSIBWO. Based on these experimental results, MSIBWO demonstrates superior convergence accuracy and robustness compared to other algorithms.

For the multimodal benchmark functions (F5-F8), MSIBWO also achieves outstanding performance. For instance, in F5, the MSIBWO algorithm attains the same ${\text{f}}_{\min}$ and average value as the BWO algorithm, indicating comparable convergence accuracy. However, MSIBWO demonstrates a lower Std, reflecting enhanced stability in reaching the optimal solution. For F6, the test results of the MSIBWO, BWO, AOA, SSA, and CPO algorithms are identical. The convergence accuracy and robustness of the five algorithms are equivalent, tying for first place. For F7, compared to the other six algorithms, the MSIBWO algorithm achieves a ${\text{f}}_{\min}$ and average value closer to the target value (see Table 1), with the smallest Std, demonstrating a significant competitive advantage in convergence accuracy and robustness. For F8, the ${\text{f}}_{\min}$ obtained by the MSIBWO and SSA algorithms are equal; however, the MSIBWO algorithm achieves the smallest average value and Std compared to the other six algorithms.

#### 2.3.2 Friedman test.

At the same time, we conducted a Friedman test to analyze the algorithm’s scalability using statistical methods [37]. Table 4 presents the results of the Friedman test, which indicate that the MSIBWO algorithm ranks first for all benchmark functions except F7. This suggests the algorithm’s consistent performance across a range of optimization problems, with the exception of specific cases.

**Table 4:** The results of Friedman test.

Function	Algorithm	Average rank	Total rank
*F*_1_(*x*)	MSIBWO	1.0000	**1**
	BWO	2.0000	2
	GWO	7.0000	7
	WOA	5.0000	5
	AOA	4.7000	4
	SSA	5.1333	6
	CPO	3.1667	3
*F*_2_(*x*)	MSIBWO	1.5000	**1**
	BWO	2.8000	2
	GWO	5.3000	6
	WOA	5.9667	3
	AOA	1.5000	**1**
	SSA	2.8667	4
	CPO	3.3333	5
*F*_3_(*x*)	MSIBWO	1.0000	**1**
	BWO	2.0333	2
	GWO	5.8000	6
	WOA	7.0000	7
	AOA	4.7000	5
	SSA	4.4000	4
	CPO	3.0667	3
*F*_4_(*x*)	MSIBWO	1.0000	**1**
	BWO	2.0333	2
	GWO	5.3667	5
	WOA	7.0000	7
	AOA	5.3333	6
	SSA	3.8667	4
	CPO	3.2000	3
*F*_5_(*x*)	MSIBWO	1.0000	**1**
	BWO	3.9667	2
	GWO	6.9667	4
	WOA	4.1667	3
	AOA	3.9667	2
	SSA	3.9667	2
	CPO	3.1667	2
*F*_6_(*x*)	MSIBWO	2.9333	**1**
	BWO	2.9333	**1**
	GWO	3.4000	3
	WOA	2.9333	**1**
	AOA	2.9333	**1**
	SSA	2.9333	**1**
	CPO	5.8667	4
*F*_7_(*x*)	MSIBWO	2.6333	2
	BWO	3.8667	4
	GWO	4.7333	5
	WOA	5.6667	6
	AOA	6.3333	7
	SSA	1.4000	**1**
	CPO	3.3667	3
*F*_8_(*x*)	MSIBWO	1.6333	**1**
	BWO	4.0333	3
	GWO	4.7333	4
	WOA	5.4000	5
	AOA	6.8000	6
	SSA	2.7000	2
	CPO	2.7000	2

**Fig 2:**
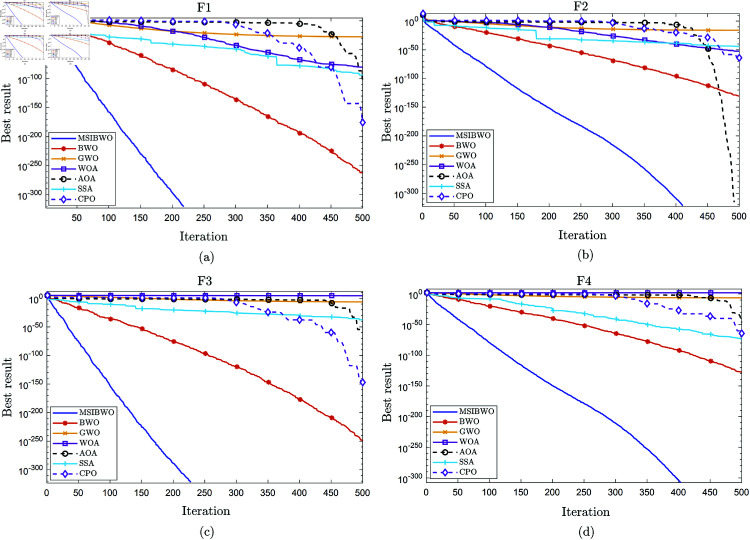
Convergence curves for unimodal function test. (a): Convergence curves of seven algorithms for F1. (b): Convergence curves of seven algorithms for F2. (c): Convergence curves of seven algorithms for F3. (d): Convergence curves of seven algorithms for F4.

**Fig 3:**
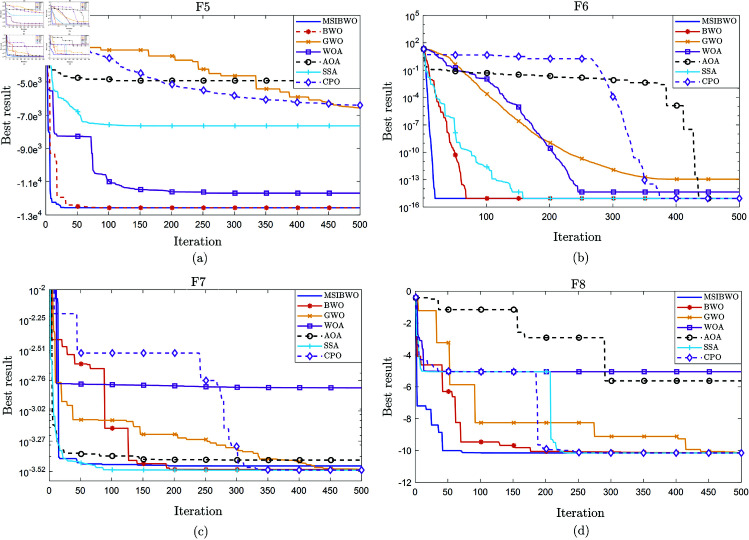
Convergence curves for multimodal function test. (a): Convergence curves of seven algorithms for F5. (b): Convergence curves of seven algorithms for F6. (c): Convergence curves of seven algorithms for F7. (d): Convergence curves of seven algorithms for F8.

Figs 2 and 3 illustrate the convergence behavior of seven algorithms for the unimodal benchmark functions (F1-F4) and the multimodal benchmark functions (F5-F8), respectively.

As shown in Fig 2, it is clear that the MSIBWO algorithm achieves strong convergence speed and optimization capability across the unimodal benchmark functions. In contrast, Fig 3 shows that for F7, although the MSIBWO algorithm demonstrates rapid convergence speed, its convergence precision is surpassed by SSA, BWO, and GWO. However, for F5, F6, and F8, MSIBWO not only converges faster than the other six algorithms but also achieves higher convergence precision, underscoring its superior performance in these cases.

In summary, while the MSIBWO algorithm exhibits suboptimal performance in certain benchmark function (F7), This phenomenon may be attributed to the characteristics of the function itself and the structure of the algorithm. Analysis reveals that F7, composed of multiple fractional terms, exhibits strong nonlinearity and multimodality. For such functions, the algorithm requires exceptionally strong global search capabilities. Although the MSIBWO algorithm improves population diversity during the initialization phase, its dynamic adaptive weight mechanism may overly emphasize local exploitation during the later stages of iteration, making it prone to getting trapped in local optima within high-dimensional nonlinear search spaces.

More importantly, Wolpert and Macready [38] proposed the “No Free Lunch Theorem” in 1997, which states that an algorithm that performs well on one type of problem may perform poorly on another [39]. Although the performance of MSIBWO on F7 has room for improvement, overall, its performance across all benchmarks is superior to the other six algorithms. Future research will focus on optimizing the algorithm for ultra-high-dimensional problems to further enhance its generalizability. These results validate the effectiveness of the multi-strategy improvements proposed in this paper, which significantly enhance the algorithm’s convergence speed, convergence accuracy, and global search capability.

#### 2.3.3 Computational complexity analysis.

To strengthen the persuasiveness of the experimental results, we also analyzed the computational complexity of the MSIBWO algorithm. Computational complexity is a critical metric for evaluating algorithm performance. It can be divided into two parts: time complexity and space complexity

The time complexity of the MSIBWO algorithm is the same as that of the BWO algorithm, consisting of three main processes: algorithm initialization, fitness value calculation, and beluga whale position updates [25]. By incorporating the Tent chaotic mapping and OBL (Opposition-Based Learning) strategies into the initialization phase, the algorithm’s initialization process is improved. Literature [31] demonstrates that the Tent chaotic mapping does not alter the original initialization time complexity, and literature [29] shows that introducing the OBL strategy into the GWO algorithm does not increase its time complexity. Therefore, the time complexity of the initialization phase in the MSIBWO algorithm is consistent with that of the BWO algorithm, expressed as O(n×d), where *n* is the population size and *d* is the dimensionality of the population.

The exploration and exploitation phases of the algorithm have a time complexity of $O\left(\left(n\times d\right)\times T_{\max}\right)$, where $T_{\max}$ is the maximum number of iterations. Due to the effect of the whale fall probability and balance factors during the whale fall phase, the time complexity is approximately $O\left(\left(n\times d\right)\times 0.1T_{\max}\right)$. Thus, the overall time complexity of the MSIBWO algorithm is $O\left(\left(n\times d\right)\times \left(1\text{ + }0.1T_{\max}\right)\right)$. Compared to the original BWO algorithm, the MSIBWO algorithm does not increase the time complexity. Additionally, its time complexity is of the same order of magnitude as other commonly used optimization algorithms (see Table 5). Furthermore, the space complexity of all these algorithms is identical, the initialization overall can be considered as the maximum amount of space occupied by the optimization method at any time [40].

**Table 5:** Comparison of computational complexity of algorithms.

Algorithm	Time complexity	Space complexity
GWO	$O\left(\left(n\times d\right)\times T_{\max}\right)$	O(n×d)
WOA	$O\left(n\times \left(1\text{+}d\times T_{\max}\right)\right)$	O(n×d)
AOA	$O\left(n\times \left(1\text{+}d\times T_{\max}\right)\right)$	O(n×d)
SSA	$O\left(\left(n\times d\right)+\left(n\times \left(d+1\right)\right)\times T_{\max}\right)$	O(n×d)
CPO	$O\left(\left(n\times d\right)+n\times T_{\max}\times \left(C\times d\right)\right)$	O(n×d)
BWO	$O\left(\left(n\times d\right)\times \left(1\text{+ }0.1T_{\max}\right)\right)$	O(n×d)
MSIBWO	$O\left(\left(n\times d\right)\times \left(1\text{+ }0.1T_{\max}\right)\right)$	O(n×d)

In conclusion, without increasing computational complexity, the MSIBWO algorithm effectively balances global and local search capabilities by improving the initialization, exploration, and exploitation phases. As a result, under the same number of iterations, the MSIBWO algorithm converges more quickly to the global optimal solution.

## 3 Establishment of mathematical model

### 3.1 Model of electrohydraulic servo actuator

**Fig 4:**
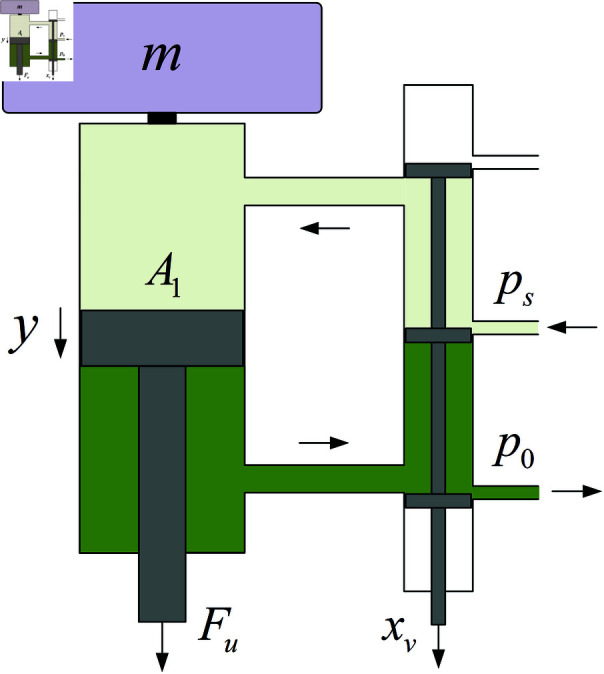
Model of electrohydraulic servo actuator.

The electrohydraulic servo actuator is modeled as shown in Fig 4. $x_{v}$ is the displacement of the servo valve spool. *y* is the displacement of the piston. *F*_*u*_ is the output actuating force of the electrohydraulic servo actuator. *p*_*s*_ is the supply pressure to the servo valve. *p*_0_ is the return pressure from the servo valve, and *A*_1_ is the cross-sectional area of the rodless chamber piston in the hydraulic cylinder.

Fig 4 shows that the force output by the electrohydraulic servo actuator is controlled by adjusting the displacement of the servo valve spool. The spool displacement of the servo valve and the input signal can be equivalent to a first-order inertial link.

$${\dot{x}}_{v}=\frac{1}{\tau }\left(-x_{v}+ku\right)$$ (12)

where, τ represents the time constant, *k* denotes the gain of the servo valve, and *u* represents the input signal.

The nonlinear mathematical model of the electrohydraulic servo actuator is expressed as follows [41]:

$$F_{u}=A_{1}p_{L}$$ (13)

$${\dot{p}}_{L}=\frac{2\beta_{e}}{V_{t}}\left(C_{d}x_{v}\sqrt{\frac{p_{s}-sigm\left(x_{v}\right)p_{L}}{\rho }}-C_{t}p_{L}-A_{1}y\right)$$ (14)

where, *p*_*L*_ is the pressure difference between the two cavities of the actuator hydraulic cylinder, $\beta_{e}$ is the oil elastic modulus, $V_{t}$ is the total volume of the actuator hydraulic cylinder, *C*_*d*_ is the flow coefficient, and *C*_*t*_ is the total leakage coefficient. The oil direction change of the servo valve can be represented by the *sigm* function, and the *sigm* function expression is as follows:

$$sigm\left(x_{v}\right)=\frac{1-e^{-a_{i}x_{v}}}{1+e^{-a_{i}x_{v}}}$$ (15)

where

$$sigm\left(x_{v}\right)=\left\{\begin{array}{c} 1\\ 0\\ -1 \end{array}\begin{array}{cccc} & & & \begin{array}{c} if\\ if\\ if \end{array} \end{array}\begin{array}{c} \begin{array}{c} a_{i}x_{v}\to \infty \\ a_{i}x_{v}\to 0\\ a_{i}x_{v}\to -\infty \end{array} \end{array}\right\}$$ (16)

### 3.2 Model of half-vehicle

**Fig 5:**
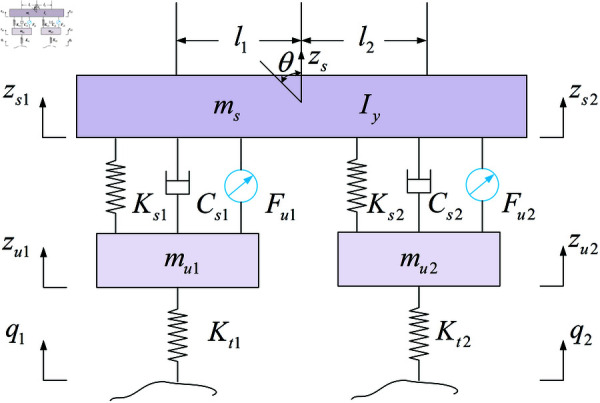
Half-vehicle model.

Building on the hydraulic servo actuator model, the half-vehicle model is established as shown in Fig 5. *m*_*s*_ is the sprung mass. *z*_*s*_ is the displacement of the sprung mass. *l*_1_ and *l*_2_ are the distance between the center line of the front axle and the center of mass of the body, respectively. *I*_*y*_ is the pitch moment of inertia of the vehicle body. θ is the pitch angle of the vehicle body. *m*_*u*_ and *m*_*u*2_ are the unsprung mass of the front suspension and rear suspension, respectively. *z*_*s*1_ and *z*_*s*2_ are the sprung mass displacement of the front suspension and rear suspension, respectively. *z*_*u*1_ and *z*_*u*2_ are unsprung mass displacements of the front suspension and the rear suspension, respectively. *q*_1_ and *q*_2_ are the road input signal. *K*_*s*1_ and *K*_*s*2_ are the stiffness coefficient of the front suspension and rear suspension, respectively.*C*_*s*1_ and *C*_*s*2_ are the damping coefficient of the front suspension and rear suspension, respectively. *K*_*t*1_ and *K*_*t*2_ are the stiffness coefficients of the front tire and the rear tire, respectively. *F*_*u*1_ and *F*_*u*2_ are the active force output of the front and rear electrohydraulic servo actuators, respectively.

Due to the small pitch angle of the vehicle body, it can be assumed that θ≈sinθ≈tanθ. The displacement calculation formulas for the sprung mass of the front and rear suspensions are as follows:

$$\{\begin{array}{c} z_{s1}=z_{s}-l_{1}\theta \\ z_{s2}=z_{s}+l_{2}\theta \end{array}$$ (17)

The dynamic equations of the half-vehicle model are given by:

$$\{\begin{array}{c} m_{s}{\ddot{z}}_{s}=\sum_{i=1}^{2}\left(F_{ksi}+F_{csi}+F_{ui}\right),i=1,2\\ m_{u1}{\ddot{z}}_{u1}=-\left(F_{ks1}+F_{cs1}+F_{u1}\right)+F_{kt1}\\ m_{u2}{\ddot{z}}_{u2}=-\left(F_{ks2}+F_{cs2}+F_{u2}\right)+F_{kt2}\\ I_{y}\theta =-\left(F_{ks1}+F_{cs1}+F_{u1}\right)l_{1}+\left(F_{ks2}+F_{cs2}+F_{u2}\right)l_{2} \end{array}$$ (18)

where, *F*_*ksi*_ is the restoring force generated by the deformation of the suspension spring. *F*_*csi*_ is the damping force produced by the suspension damper, and *F*_*kti*_ is the force exerted by the tire on the unsprung mass. Their expressions are as follows:

$$F_{ksi}=K_{si}\left(z_{ui}-z_{si}\right)+\varepsilon K_{si}{\left(z_{ui}-z_{si}\right)}^{3}$$ (19)

$$F_{csi}=C_{si}-C_{1}\left|{\dot{z}}_{ui}-{\dot{z}}_{si}\right|+C_{2}\sqrt{\left|{\dot{z}}_{ui}-{\dot{z}}_{si}\right|}\text{s}gn\left({\dot{z}}_{ui}-{\dot{z}}_{si}\right)$$ (20)

$$F_{kti}=K_{t}\left(q_{i}-z_{ui}\right)$$ (21)

## 4 The design of MSIBWO-FOPID

### 4.1 Fractional Order PID Controller

(FOPID) In control systems, the Grünwald-Letnikov (GL) definition is often used to describe fractional calculus. The expression is as follows:

aDtαf(t)=limh→0h−α∑j=0[t−ah](−1)j(αj)f(t−jh) (22)

where, aDtα is the fractional calculus operator, α is the fractional order, *t*,*a* is the upper and lower limits of calculus, *f*(*t*) is the calculus function, and [•] is the integer operator.

The parameters $K_{p},K_{i},K_{d}$ in FOPID represent the proportional, integral, and differential gains, respectively. There are also two parameters λ and μ which are the integral order and differential order respectively, both of which are positive real numbers. The transfer function of FOPID [15] is expressed as follows:

$$G_{c}=K_{p}\text{ + }\frac{K_{i}}{s^{\lambda }}+K_{d}s^{\mu }$$ (23)

### 4.2 Optimize the parameters of FOPID

**Fig 6:**
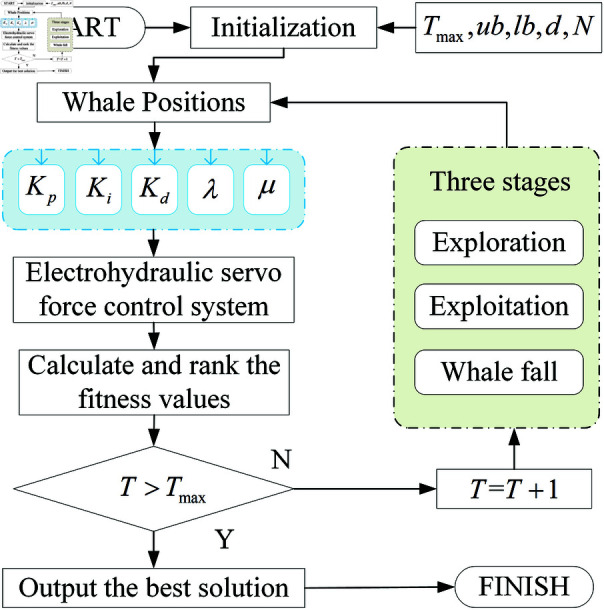
The FOPID parameter optimization flow chart based on the MSIBWO algorithm.

The FOPID parameter optimization flow chart is based on the MSIBWO algorithm as shown in Fig 6. First, the initialization parameters are determined to initialize the population. Next, for electrohydraulic servo control systems, the performance is generally evaluated using the error integration criterion. To improve the dynamic performance of the system, the Integral of Squared Error (ISE) is commonly used as the objective function [15]:

$$J=\int_{0}^{Ts}te{\left(t\right)}^{2}dt$$ (24)

where, *T*_*s*_ is the simulation time. The algorithm proceeds through three stages for position updating and iterative optimization. Finally, it outputs the optimal fitness value and the corresponding controller parameters.

## 5 Results and discussion

Using the electrohydraulic servo actuator and the half-vehicle as research subjects, the corresponding simulation models are constructed in MATLAB/Simulink to compare the dynamic performance of the system under MSIBWO-FOPID, BWO-FOPID, and PID control.

### 5.1 Simulation analysis of the electrohydraulic servo actuator

**Fig 7:**
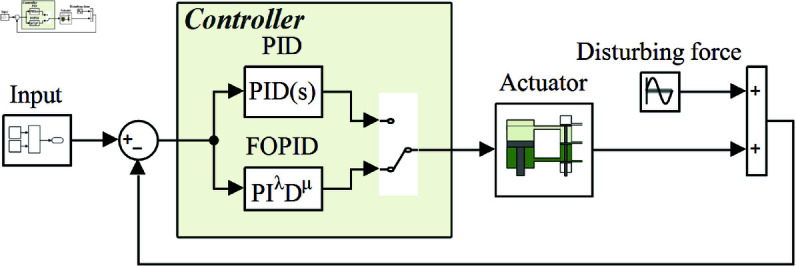
The control system for the electrohydraulic servo actuator.

The control system of the electrohydraulic servo actuator is developed using MATLAB/Simulink, as illustrated in Fig 7. The corresponding system parameters are provided in Table 6. To evaluate the dynamic force control performance, a comparative analysis is conducted under three different control schemes: MSIBWO-FOPID, BWO-FOPID, and PID control.

**Table 6:** The parameters of the electrohydraulic servo actuator.

Parameter	Value	Parameter	Value
*A*_1_ (${\text{m}}^{2}$)	$1.3\times 10^{-3}$	ρ (${\text{kg/m}}^{3}$)	850
*C*_*d*_	$1.57\times 10^{-3}$	$V_{t}$ (${\text{mm}}^{3}$)	$8.51\times 10^{-4}$
τ (s)	$3.33\times 10^{-2}$	$\beta_{e}$ (Pa)	$7.5\times 10^{8}$
*p*_*s*_ (Pa)	$2.1\times 10^{7}$	*C*_*t*_ (${\text{mm}}^{3}/\text{s}\cdot \text{Pa}$)	$3.0\times 10^{-11}$
*k* (-)	$6.3\times 10^{-6}$	$\alpha_{i}$ (-)	$2\times 10^{3}$

**Fig 8:**
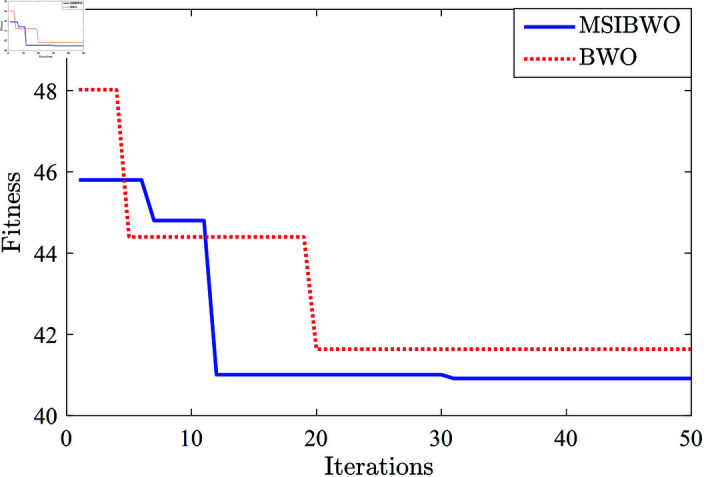
The iterative curves of the algorithms.

Based on the results of the previous benchmark function tests and the Friedman test, the MSIBWO algorithm demonstrates superior comprehensive performance. This subsection provides a detailed comparison of the MSIBWO and BWO algorithms in optimizing the controller parameters. The lower bounds and the upper bounds are designed as lb=[0,0,0,0,0] and ub=[1,1,1,1,1.5]. Fig 8 shows the iterative curves of the algorithms.

As shown in Fig 8, the MSIBWO algorithm consistently outperforms the BWO algorithm. The key observations are summarized as follows:

1. During the initialization phase, the MSIBWO algorithm exhibits a smaller fitness value. This indicates that its population initialization strategy generates higher-quality whale positions, leading to initial solutions that are closer to the global optimum compared to the BWO algorithm.
2. Both the convergence speed and accuracy of the MSIBWO algorithm surpass those of the BWO algorithm. The enhanced exploration strategy significantly broadens the search range during the early stages, improving the algorithm’s global search capability. Furthermore, the incorporation of dynamic adaptive weights during the exploitation phase accelerates the convergence process.

In conclusion, the MSIBWO algorithm achieves superior performance in terms of both convergence efficiency and optimization accuracy compared to the BWO algorithm.

Table 7 shows the parameter values corresponding to the three controllers.

**Table 7:** The values corresponding to the three controllers.

Controller parameter	Controller		
	PID	BWO-FOPID	MSIBWO-FOPID
*K*_*p*_	0.131	0.2659	0.3050
*K*_*i*_	0.0032	0.3668	0.1315
*K*_*d*_	0.0038	0.0180	0.2062
λ	-	0.4432	0.1042
μ	-	1.0157	1.0969

#### 5.1.1 Step signal.

**Fig 9:**
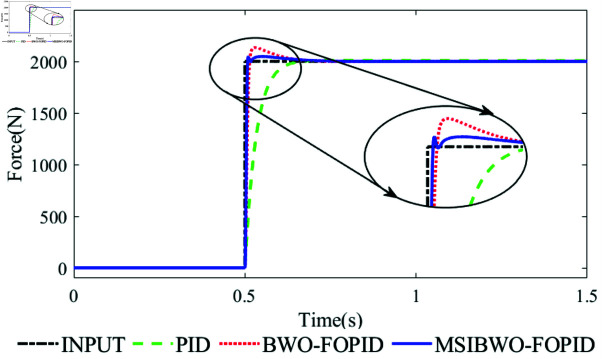
Dynamic force-response curve of the system under step signal.

A step signal is used as the input to evaluate the force-tracking performance of the electrohydraulic servo actuator with different controllers. The force-response curves are presented in Fig 9, while Table 8 summarizes the key performance indicators of the system with different controllers, indicators include: overshoot, rise time and root mean square errors (RMSE).

**Table 8:** The system response performance indicators.

Performance Indicator	Controller		
	MSIBWO-FOPID	BWO-FOPID	PID
Overshoot(%)	2.44	6.73	**0.2770**
Rise time(*ms*)	**6.30**	10.50	73.50
RMSE	**0.8354**	1.4231	37.9601

As shown in Fig 9, the maximum overshoot of the system for MSIBWO-FOPID, BWO-FOPID, and PID controllers are 2.44%, 6.73%, and 0.51%, respectively. Notably, the PID controller exhibits minimal overshoot but has the longest rise time compared to the other two controllers. Regarding transient performance, the rise times of the system for MSIBWO-FOPID and BWO-FOPID controllers are 6.3 *ms* and 10.5 *ms*, respectively, which are both considerably shorter than the 73.5 *ms* rise time observed for the PID controller. For steady-state performance, Table 8 highlights that the root mean square errors (RMSE) under MSIBWO-FOPID, BWO-FOPID, and PID controllers are 0.8354, 1.4231, and 37.9601, respectively, indicating a substantial advantage of MSIBWO-FOPID in reducing steady-state errors.

To eliminate the possibility of randomness in the conclusion that "the control performance of FOPID parameters tuned by the MSIBWO algorithm is superior to BWO-FOPID and traditional PID," and to enhance the credibility of the results, 10 parameter tuning tasks were conducted, followed by step response tests (Table 9).

**Table 9:** Test results for step response.

Controller	Overshoot(%)	Rise time(*ms*)	RMSE
MSIBWO-FOPID	2.44	6.30	0.8354
	2.61	6.12	0.8437
	2.39	5.84	0.8329
	2.97	7.03	0.8479
	2.73	6.45	0.8432
	2.56	6.17	0.8314
	2.34	5.92	0.8193
	2.88	6.67	0.8572
	3.13	7.18	0.8638
	2.91	6.73	0.8541
BWO-FOPID	6.73	10.50	1.4231
	6.57	10.11	1.4092
	6.91	10.86	1.4427
	6.62	10.24	1.4126
	6.83	10.58	1.4353
	6.96	10.77	1.4432
	7.05	11.36	1.4691
	6.76	10.48	1.4239
	7.02	11.03	1.4403
	6.69	10.59	1.4234
PID	0.51	73.50	37.9601
	0.48	70.13	36.4724
	0.43	68.58	35.8147
	0.56	73.92	38.0026
	0.53	74.56	38.6458
	0.47	69.19	36.0915
	0.67	77.93	40.1863
	0.59	73.73	38.4119
	0.54	74.35	38.5345
	0.49	73.64	37.9581

From the analysis of Table 9, the data exhibits characteristics such as multiple independent samples, small sample sizes, and unknown data distribution. Therefore, the Kruskal-Wallis test, a non-parametric method, was selected. The significance analysis was performed using SPSS software, and the test results are shown in Table 10.

**Table 10:** Results of Kruskal-Wallis test under step signal.

Null Hypothesis	Significance	Decision
The distribution of overshoot is the same across controller categories	<0.001	Reject the null hypothesis
The distribution of rise time is the same across controller categories	<0.001	Reject the null hypothesis
The distribution of RMSE is the same across controller categories	<0.001	Reject the null hypothesis

From Table 10, the significance levels of the three performance metrics for different control systems are all far below the threshold of 0.05, indicating significant differences. Combining Tables 8 and 9, it is evident that the MSIBWO-FOPID control system achieves the best overall force-tracking performance.

In conclusion, the electrohydraulic servo actuator controlled by MSIBWO-FOPID achieves the smallest force-tracking error, superior transient response, and the highest force-tracking accuracy, making it the most effective control strategy among the three.

#### 5.1.2 Sine signal.

A sine signal is applied as the input to evaluate the robustness and disturbance rejection capabilities of the system under different controllers.

**(1) System Robustness**. During vehicle operation, various road conditions require the electrohydraulic servo actuator to adaptively output forces. This demands that the controllers exhibit strong robustness to maintain performance under varying conditions.

**Fig 10:**
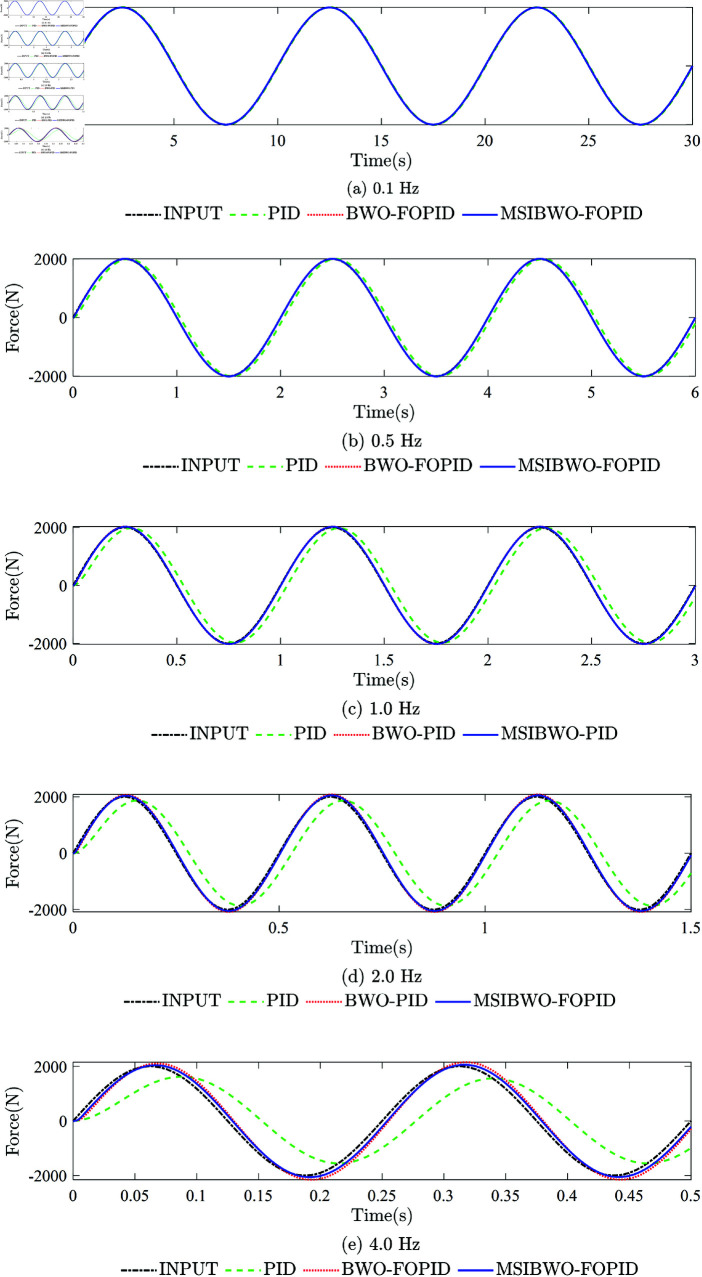
The force-response curves of the system under different frequency sine signals. (a) The force-response curve of the system at a sine signal frequency of 0.1 Hz. (b) The force-response curve of the system at a sine signal frequency of 0.5 Hz. (c) The force-response curve of the system at a sine signal frequency of 1.0 Hz. (d) The force-response curve of the system at a sine signal frequency of 2.0Hz. (e) The force-response curve of the system at a sine signal frequency of 4.0 Hz.

To assess robustness, the input signal is configured with a constant amplitude of 2000 N and varying frequencies (0.1 Hz, 0.5 Hz, 1.0 Hz, 2.0 Hz, and 4.0 Hz). The corresponding force-response curves are presented in Fig 10. The performance indicators (RMSE) are summarized in Table 11. (The smaller the RMSE, the higher the tracking accuracy of the system).

**Table 11:** The RMSE for the system under different frequency sine signals.

Frequency ( Hz)	Controller		
	MSIBWO-FOPID	BWO-FOPID	PID
0.1	**0.0115**	0.0173	0.3143
0.5	**0.2738**	0.3399	0.5723
1.0	**0.9957**	1.3336	3.7570
2.0	**2.5657**	4.4470	14.9102
4.0	**3.7498**	10.5927	64.1120

**As shown in** Fig 10**(a), at a frequency of 0.1 Hz**, all three controllers effectively track the input signal. However, the MSIBWO-FOPID controller demonstrates superior force-tracking performance, reducing RMSE by 33.53% and 96.34% compared to BWO-FOPID and PID, respectively.

**At 0.5 Hz** (Fig 10**(b))**, force-tracking performance begins to decline for all controllers. Despite this, the MSIBWO-FOPID controller achieves the best performance, with RMSE reductions of 19.44% and 52.16% relative to BWO-FOPID and PID, respectively.

**At 1.0 Hz** (Fig 10**(c))**, the tracking performance deteriorates further. Yet, the MSIBWO-FOPID controller still outperforms the others, reducing RMSE by 25.34% and 73.50% compared to BWO-FOPID and PID, respectively.

**At 2.0 Hz** (Fig 10**(d))**, the PID controller exhibits the highest force-tracking error, followed by BWO-FOPID, while MSIBWO-FOPID achieves the lowest error. Table 11 indicates that MSIBWO-FOPID reduces RMSE by 42.30% and 82.79% compared to BWO-FOPID and PID, respectively.

**At 4.0 Hz** (Fig 10**(e))**, the tracking performance under PID deteriorates significantly, while MSIBWO-FOPID continues to maintain acceptable performance, reducing RMSE by 64.60% and 94.15% relative to BWO-FOPID and PID, respectively.

Similarly, to rule out randomness in the conclusion that "the control performance of FOPID parameters tuned by the MSIBWO algorithm is superior to BWO-FOPID and traditional PID," further reliability tests were conducted. For each controller, force-response tests were performed under sinusoidal input signals of various frequencies, with simulation results presented in Table 12.

**Table 12:** Test results for sine response.

Frequency (Hz)	MSIBWO-FOPID	BWO-FOPID	PID
0.1	0.0114, 0.0115, 0.0114, 0.0115,	0.0172, 0.0170, 0.0174, 0.0170,	0.3156, 0.3033, 0.2978, 0.3160,
	0.0115, 0.0114, 0.0113, 0.0116,	0.0173, 0.0174, 0.0177, 0.0172,	0.3216, 0.3001, 0.3342, 0.3195,
	0.0117, 0.0116	0.0174, 0.0172	0.3205, 0.3155
0.5	0.2715, 0.2743, 0.2701, 0.2743,	0.3372, 0.3341, 0.3423, 0.3345,	0.5754, 0.5528, 0.5425, 0.5759,
	0.2738, 0.2709, 0.2669, 0.2780,	0.3399, 0.3423, 0.3485, 0.3374,	0.5859, 0.5502, 0.6121, 0.5840,
	0.2797, 0.2771	0.3420, 0.3373	0.5856, 0.5752
1.0	0.9873, 0.9975, 0.9822, 0.9975,	1.3209, 1.3079, 1.3405, 1.3088,	3.7350, 3.5879, 3.5203, 3.7408,
	0.9957, 0.9842, 0.9697, 1.0100,	1.3336, 1.3405, 1.3653, 1.3223,	3.8064, 3.5651, 3.9698, 3.7882,
	1.0168, 1.0063	1.3391, 1.3220	3.7989, 3.7340
2.0	2.5440, 2.5709, 2.5331, 2.5709,	4.4072, 4.3645, 4.4721, 4.3671,	14.786, 14.207, 13.943, 14.808,
	2.5657, 2.5391, 2.5006, 2.6071,	4.4470, 4.4721, 4.5555, 4.4121,	15.068, 14.114, 15.713, 14.985,
	2.6243, 2.5979	4.4670, 4.4117	15.021, 14.782
4.0	3.718, 3.757, 3.707, 3.757,	10.502, 10.401, 10.657, 10.414,	63.61, 61.12, 59.97, 63.68,
	3.750, 3.709, 3.652, 3.805,	10.593, 10.657, 10.855, 10.508,	64.78, 60.66, 67.54, 64.46,
	3.831, 3.792	10.642, 10.507	64.62, 63.60

Statistical significance testing was conducted based on the results in Table 12. The Kruskal-Wallis test was again used, and significance analyses were performed for each frequency using SPSS software. The results are shown in Table 13.

**Table 13:** Results of Kruskal-Wallis test under sine signals.

Frequency	Null Hypothesis	Significance	Decision
0.1(Hz)	The distribution of RMSEis the same	<0.001	Reject the null hypothesis
0.5(Hz)	across controller categories	<0.001	Reject the null hypothesis
1.0(Hz)		<0.001	Reject the null hypothesis
2.0(Hz)		<0.001	Reject the null hypothesis
4.0(Hz)		<0.001	Reject the null hypothesis

From Table 13, it can be observed that the RMSE of the system under the three controllers shows significant differences across all sine input frequencies. Combining Tables 11 and 12, it is clear that the MSIBWO-FOPID control system achieves the smallest RMSE.

In conclusion, under varying frequencies, the MSIBWO-FOPID controller consistently demonstrates the best force-tracking performance. As the sine signal frequency increases from 0.1 Hz to 4.0 Hz, the MSIBWO-FOPID controller exhibits the smallest variation in force-tracking error, highlighting its strong robustness and adaptability.

**Fig 11:**
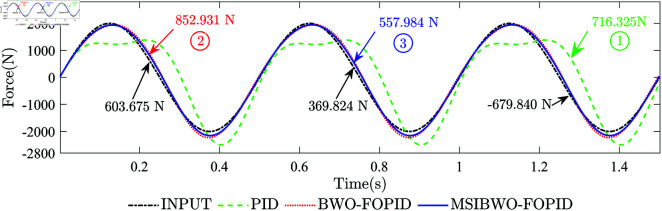
The force-response curves of the system with external disturbance forces.

**Fig 12:**
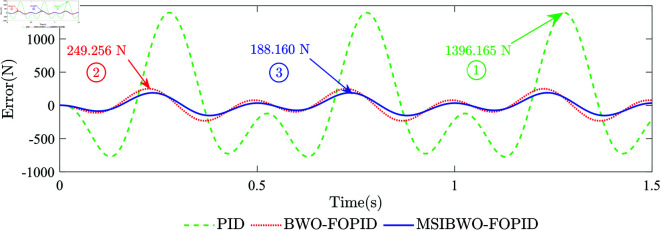
The force-tracking error curves.

**(2) System Disturbance Resistance**. During vehicle operation, external nonlinear disturbance forces can degrade the performance of the active suspension system [42]. Therefore, to enhance the performance of the active suspension system, the selected controller for the electrohydraulic servo actuator system needs to possess strong disturbance resistance. A sine signal with an amplitude of 2000 N and a frequency of 2.0 Hz is used as the input signal. Simultaneously, an external disturbance signal with an amplitude of 1000 N and a frequency of 4.0 Hz is applied to evaluate the disturbance rejection performance of different controllers. Fig 11 shows the force-response curves of the system with external disturbance forces under different controllers, while Fig 12 provides the force-tracking error curves for the different controllers.The maximum force-tracking error points under PID, BWO-FOPID, and MSIBWO-FOPID controllers are visually marked as points 1, 2, and 3, respectively, in Figs 11 and 12. The black arrows in Fig 11 indicate the input force values at these marked points. The deviation values of the two values at the marked points in Fig 11 correspond to the values in Fig 12. Table 14 presents the RMSE for the system under the different controllers.

**Table 14:** The RMSE for the system under the different controllers.

Frequency ( Hz)	Controller		
	MSIBWO-FOPID	BWO-FOPID	PID
2.0	**1.9419**	2.6822	4.2611

From Figs 11 and 12, it is evident that external disturbance forces affect the force-tracking performance of all three controllers. Among them, the MSIBWO-FOPID controller exhibits the highest tracking accuracy. Regarding the maximum force-tracking error, the marked points in Figs 11 and 12 reveal the following:

Under PID control, the maximum force-tracking error occurs at 1.2776 s (point 1), where the input force is -679.840 N, the system output force is 716.325 N, and the tracking error is 1396.165 N.

Under BWO-FOPID control, the maximum force-tracking error occurs at 0.2256 s (point 2), where the input force is 603.675 N, the system output force is 852.931 N, and the tracking error is 249.256 N.

Under MSIBWO-FOPID control, the maximum force-tracking error occurs at 0.7352 s (point 3), where the input force is 369.824 N, the system output force is 557.984 N, and the tracking error is only 188.160 N. Regarding the RMSE of force-tracking errors (Table 14), the MSIBWO-FOPID control system reduces the RMSE by 27.60% compared to BWO-FOPID and by 54.43% compared to PID control. In summary, the system under MSIBWO-FOPID control exhibits the smallest force-tracking error and the strongest anti-disturbance capability. Its force-tracking accuracy is significantly superior to that of BWO-FOPID control and PID control.

### 5.2 Simulation analysis of the half-vehicle model

**Fig 13:**
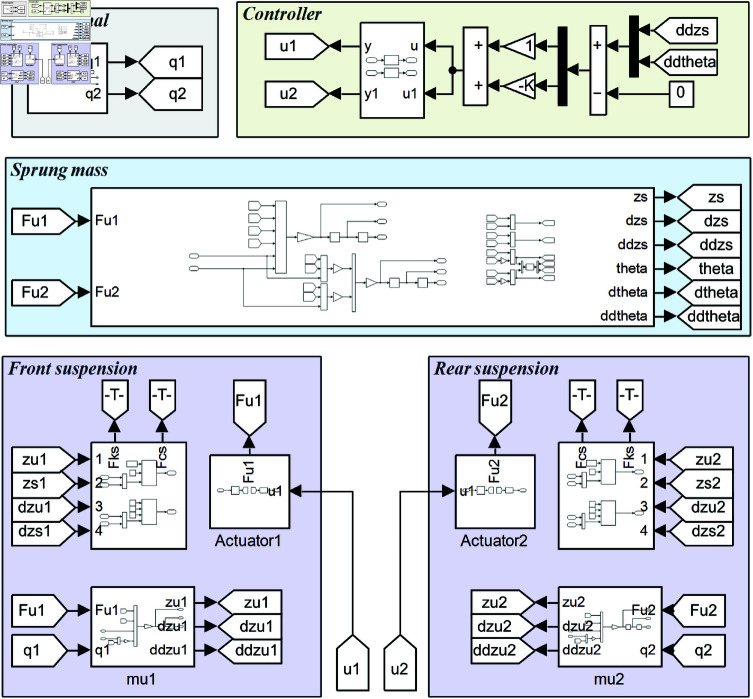
The half-vehicle model is established in MATLAB/Simulink.

In previous sections, it has been demonstrated that the MSIBWO-FOPID controller significantly enhances the force output precision of the electrohydraulic servo actuator. Building on these findings, a half-vehicle model is employed to evaluate the ride comfort performance of MSIBWO-FOPID, BWO-FOPID, and PID controllers. Key evaluation metrics include vertical acceleration and pitch angle acceleration, which are widely used indicators of ride comfort. The half-vehicle model, established in MATLAB/Simulink (Fig 13), incorporates the parameters listed in Table 15.

**Table 15:** The parameters of the half-vehicle model.

Parameter	Value	Parameter	Value
*m*_*s*_ (kg)	750	*l*_1_ (m)	1.4
$\mu_{i}$ (kg)	43.157	*l*_2_ (m)	1.65
*I*_*y*_ (N/m)	1500	*C*_*si*_ (N·s/m)	850
*K*_*si*_ (N/m)	14036	*C*_1_ (N·s/m)	350.02
*K*_*ti*_ (N/m)	241340	*C*_2_ (N·s/m)	350.1
ε (-)	80.01	*C*_*t*_ (${\text{mm}}^{3}/\text{s}\cdot \text{Pa}$)	$3.0\times 10^{-11}$

To verify the effectiveness of the controller, it is necessary to establish a road surface model that closely approximates real-world conditions. In this study, the filtered white noise method is adopted to generate random road signals. This method has the advantages of low computational complexity and accurately reflecting actual road conditions. The road roughness is described using the road power spectral density (PSD), which is expressed as follows:

$$\dot{q}(t)=-2\pi n_{c}v_{c}2q(t)+2\pi n_{0}\sqrt{G_{q}(n_{0})v_{c}}w(t)$$ (25)

where, *n*_0_ is the reference spatial frequency, *n*_*c*_ is the lower cutoff spatial frequency, *w*(*t*) is Gaussian white noise, *G*_*q*_ is the road roughness coefficient, $v_{c}$ is the vehicle speed. The parameters are set based on real road conditions and ISO 8608 [43]:

The reference spatial frequency *n*_0_ is set to $0.1{\text{m}}^{-1}$. To filter out low-frequency signals that have minimal impact on ride comfort, the lower cutoff frequency *n*_*c*_ is set to $0.05{\text{m}}^{-1}$. According to the road roughness classification standard provided by the International Organization for Standardization (ISO), typical urban or suburban road surfaces correspond to Class C, with a road roughness coefficient *G*_*q*_ of 2.56 × $10^{-4}m^{3}$. The vehicle speed $v_{c}$ is set to 36*km*/*h* to simulate typical driving conditions.

**Fig 14:**
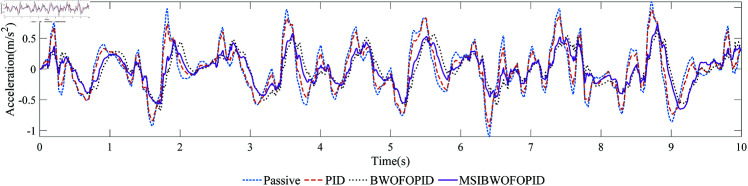
The vertical acceleration curve of the vehicle body.

**Fig 15:**
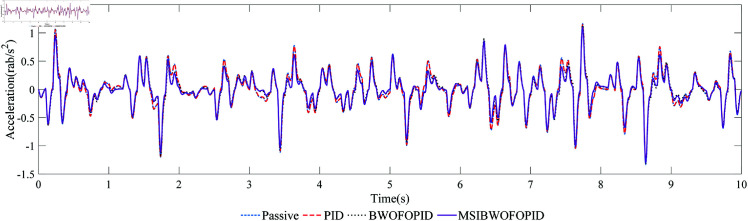
The pitch acceleration curve of the vehicle body.

Under the excitation of random road surface signal, Figs 14 and 15 show the comparison curves of the vehicle body’s ride comfort under different controllers. Table 16 compares the ride comfort indicators, *R*1 and *R*2 represent the root mean square (RMS) of the vertical and pitch accelerations of the vehicle body, respectively.

**Table 16:** The ride comfort indicators.

Performance indicator	Control strategy			
	MSIBWO-FOPID	BWO-FOPID	PID	No control
*R*1	**0.2620**	0.2771	0.3606	0.3975
*R*2	**0.2833**	0.2927	0.3163	0.3204

Under random road excitation, all three controllers effectively suppress vertical and pitch accelerations, thereby improving ride comfort. As shown in Fig 14, Fig 15, and Table 16, the MSIBWO-FOPID controller achieves superior performance compared to the other methods. Specifically, the vertical acceleration of the vehicle body is reduced by 10.2%, 43.5%, and 51.7% under PID, BWO-FOPID, and MSIBWO-FOPID, respectively. Similarly, the pitch acceleration decreases by 1.3%, 9.5%, and 13.1% for the same methods.

These results highlight the significant advantage of MSIBWO-FOPID in improving ride comfort, making it the most effective among the three controllers. In summary, using a half-vehicle model and random road signals, the MSIBWO-FOPID controller demonstrates superior ride comfort performance, validating its effectiveness and practical applicability.

## 6 Conclusion and outlook

### 6.1 Conclusion

This study proposed an intelligent optimization control strategy The effectiveness of the strategy has been validated through comprehensive simulation analysis, with the following key contributions:

1. A novel MSIBWO algorithm was introduced and rigorously evaluated through benchmark function testing and the Friedman test. Compared to other algorithms, the MSIBWO algorithm demonstrated superior performance in terms of convergence speed and optimization accuracy.
2. A parameter optimization method for the FOPID controller was developed using the MSIBWO algorithm. When applied to the electrohydraulic servo actuator model, the MSIBWO-FOPID controller achieved higher output force precision, enhanced robustness, and improved disturbance rejection compared to BWO-FOPID and PID controllers.
3. Simulation results using a half-vehicle model under random road excitation revealed that the MSIBWO-FOPID controller significantly improved ride comfort, outperforming BWO-FOPID and PID controllers in both vertical and pitch acceleration metrics.

In the simulation tests, the proposed MSIBWO-FOPID controller demonstrated excellent control performance. When applied to real-world vehicles, the following issues need to be addressed:

1. **Hardware Analysis**. ECU Computational Requirements: First, the computational demand of the in-vehicle electronic control unit (ECU) must be analyzed. With the continuous advancements in computer hardware technology, there are already commercially available ECUs capable of supporting the execution of complex algorithms. These devices boast powerful computational capabilities and high-speed communication, providing the necessary support for the practical implementation of the MSIBWO-FOPID algorithm. Sensor Accuracy Requirements: Second, in terms of sensor accuracy, high-precision sensors can be employed. Alternatively, by integrating a Kalman filter algorithm, the output signals from lower-accuracy sensors can be processed to effectively eliminate noise and interference, thereby improving the signal-to-noise ratio (SNR).
2. **Cost and Scalability Analysis**. The use of high-end ECUs may lead to increased costs. However, the rapid pace of technological advancement and iteration is driving down the prices of high-end ECUs. Additionally, through reasonable algorithm design and optimization, the algorithm can be made lightweight, thereby reducing the performance requirements for ECUs. This not only helps control costs but also ensures compatibility with different hardware platforms, enhancing the scalability of the algorithm.

### 6.2 Outlook

Considering the time lag in existing control strategies, which rely on passive adjustments after encountering sudden changes in road surfaces, the overall performance of the vehicle is significantly constrained. Future research will focus on designing a preview-based active suspension control strategy based on road elevation. The specific research objectives are as follows:

1. Obtain road surface information ahead of the vehicle using sensors and calculate the changes in the vehicle’s pose and attitude.
2. With the goal of improving ride comfort and handling stability, determine the suspension adjustment values based on the current and predicted future vehicle attitudes. These adjustment values will be input into the MSIBWO-FOPID controller to achieve proactive pre-adjustment of the suspension system.
